# Highly Sensitive Measurement of Bio-Electric Potentials by Boron-Doped Diamond (BDD) Electrodes for Plant Monitoring

**DOI:** 10.3390/s151026921

**Published:** 2015-10-23

**Authors:** Tsuyoshi Ochiai, Shoko Tago, Mio Hayashi, Akira Fujishima

**Affiliations:** 1 Kanagawa Academy of Science and Technology, KSP Building East 407, 3-2-1 Sakado, Takatsu-ku, Kawasaki, Kanagawa 213-0012, Japan; E-Mails: pg-tago@newkast.or.jp (S.T.); pg-hayashi@newkast.or.jp (M.H.); 2 Photocatalysis International Research Center, Tokyo University of Science, 2641 Yamazaki, Noda, Chiba 278-8510, Japan; E-Mail: fujishima_akira@admin.tus.ac.jp

**Keywords:** Boron-doped Diamond (BDD) electrodes, bio-electric potentials, plant monitoring

## Abstract

We describe a sensitive plant monitoring system by the detection of the bioelectric potentials in plants with boron-doped diamond (BDD) electrodes. For sensor electrodes, we used commercially available BDD, Ag, and Pt plate electrodes. We tested this approach on a hybrid species in the genus *Opuntia* (potted) and three different trees (ground-planted) at different places in Japan. For the *Opuntia*, we artificially induced bioelectric potential changes by the surface potential using the fingers. We detected substantial changes in bioelectric potentials through all electrodes during finger touches on the surface of potted *Opuntia* hybrid plants, although the BDD electrodes were several times more sensitive to bioelectric potential change compared to the other electrodes. Similarly for ground-planted trees, we found that both BDD and Pt electrodes detected bioelectric potential change induced by changing environmental factors (temperature and humidity) for months without replacing/removing/changing electrodes, BDD electrodes were 5–10 times more sensitive in this detection than Pt electrodes. Given these results, we conclude that BDD electrodes on live plant tissue were able to consistently detect bioelectrical potential changes in plants.

## 1. Introduction

Plants have various capabilities to detect environmental factors such as atmospheric temperature, humidity, and light intensity. This becomes obvious by monitoring their bioelectric potential changes [1,2,3,4,5]. In this study, we describe a novel approach for plant monitoring which uses BDD electrodes [6,7,8,9,10] to detect electrochemical signals in plants. Our methodology builds off of previous work [1,2,3,4,5] which showed that in plants electrochemical signals change in response to changing environmental factors (e.g., light, temperature, humidity, and atmospheric pressure). We chose to specifically use BDD electrodes because they have been shown to have excellent electrochemical sensitivity [6,7,8,9] and have proven suitable for *in vivo* electrochemical detection [8,9]. For comparison, we also used commercially available Ag and Pt plate electrodes. We tested this approach on a hybrid species in the genus *Opuntia* (potted) and three different trees (ground-planted) at different places in Japan. In this time, we artificially induced bioelectric potential changes using the surface potential of the human finger for the potted *Opuntia*. On the other hand, for the ground-planted trees, we recorded not only bioelectric potential changes of the trees but also environmental factors (temperature and humidity) changes for months without replacing/removing/changing electrodes.

## 2. Experimental Section

**Figure 1:**
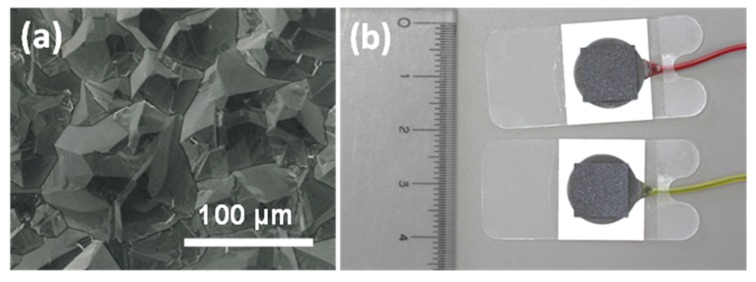
(**a**) A SEM image of the BDD electrode; (**b**) A photograph of the sensor electrodes.

Polycrystalline BDD plate electrodes (10 mm × 10 mm, Figure 1a) were purchased from Element Six Ltd. (Tokyo office, Tokyo, Japan). The Pt and Ag plate electrodes (10 mm × 10 mm) were purchased from Nilaco Corp. (Tokyo, Japan). As shown in Figure 1b, for SEM observation each electrode was attached with conductive carbon tape (Cat. No. 7311, Nisshin EM Co., Ltd., Tokyo, Japan) onto 3M™ Red Dot™ electrocardiogram (ECG) monitoring electrodes (Ag/AgCl) including a pre-attached 2269TP lead wire to be usable as a sensor electrode. The tape resistance was too low for whole circuit to affect the results of monitoring plants.

**Figure 2:**
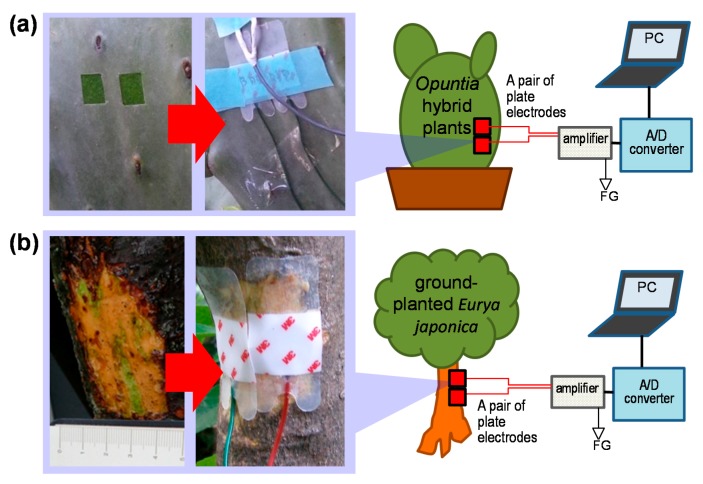
Photographs and schematics of typical experimental setup for detecting electrochemical signals for the potted *Opuntia* hybrid plants (**a**) and the ground-planted *Eurya japonica*; (**b**). FG: frame background, *i.e.*, the earth.

Figure 2 shows photographs and schematics of a typical experimental setup for detecting the electrochemical signals of plants. For the potted *Opuntia* hybrid plants (Figure 2a), we peeled the epidermis to exposed a small rectangular areas of the green phloem tissue (at least 10 mm × 10 mm to fit each electrode). We then put each pair of sensor electrodes (BDD, Pt, and Ag plate electrodes) on the areas before the green layers changed their own color to brown. Then we fixed them with plastic tape. The electrodes are fixed at the same height with 10 mm of gap.

Similarly, each pair of BDD and Pt plate electrodes was fixed onto the ground-planted trees such as *Eurya japonica* (Figure 2b). We sliced off the crude bark of the trees at approximately 1 m of height from the ground and made a small flat area of exposed green phloem tissue to fit the electrodes. After fixing the electrodes, we tied them up with plastic tape and wrapped aluminum foil around the tree to avoid rain and electromagnetic noise.

**Figure 3:**
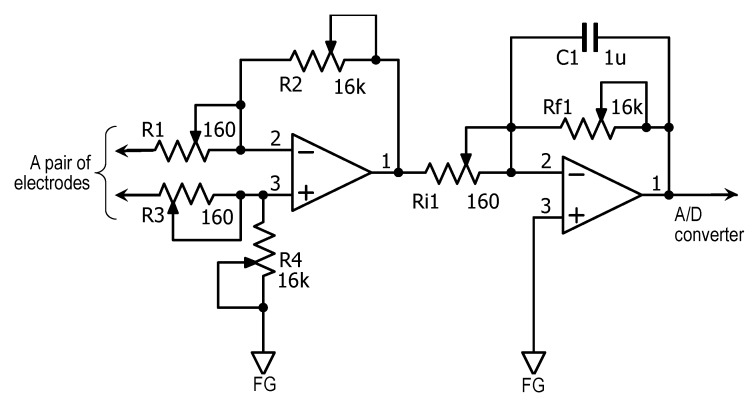
Amplifier circuit for electrochemical signals in plants.

Bioelectric potentials in the plants were collected by the pair of sensor electrodes on each individual plant and amplified by a handmade amplifier shown in Figure 3. In order to reduce instability and noise, this amplifier was constructed as a differential amplification circuit with a gain of 10,000 and a 10 Hz low pass filter. All analog signals from the plants were converted to digital signals and transferred to a personal computer through a 24-bit analog/digital converter with data logger, ADC-24 (Pico Technology Ltd., St Neots, UK), at a sampling rate of 1 sample per second (for *Opuntia* hybrid plants) or per minute (for the ground-planted trees). For *Opuntia* hybrid plants, we artificially induced bioelectric potential changes using the surface potential of a finger. On the other hand, we measured naturally induced bioelectric potential changes in the ground-planted trees.

## 3. Results and Discussion

**Figure 4:**
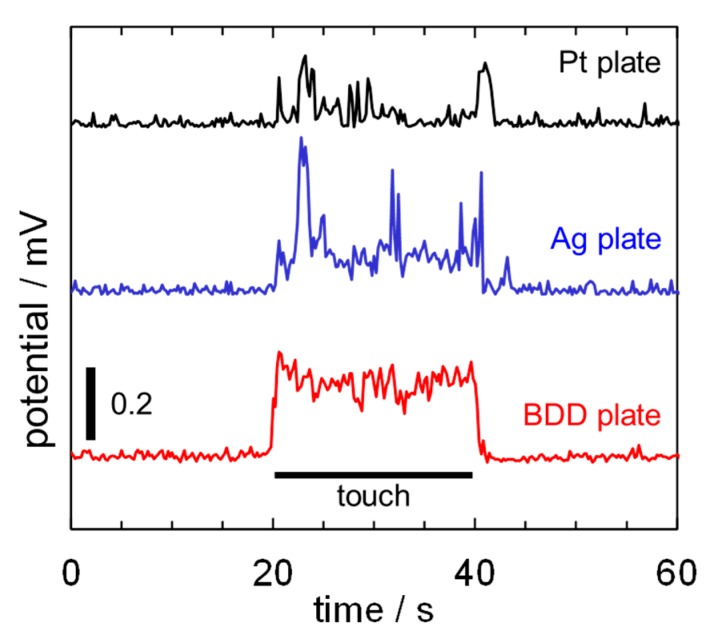
Electrochemical signals in potted *Opuntia* hybrid plants evoked by a finger touch monitored with the electrodes.

**Figure 5:**
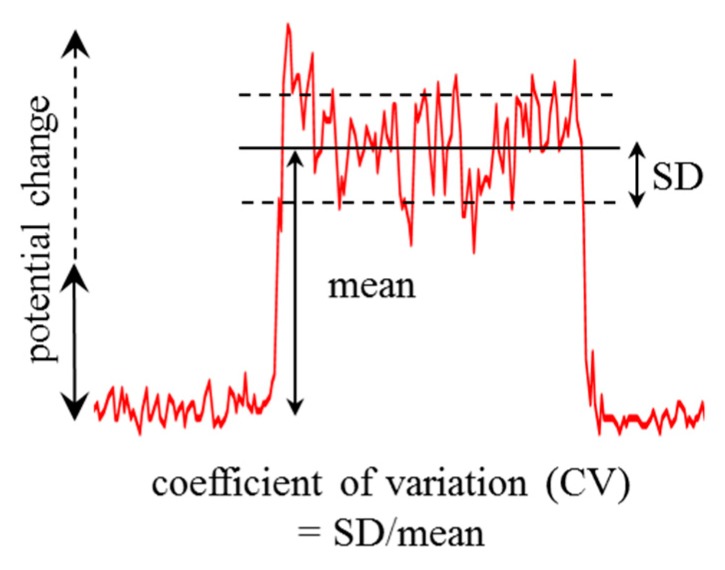
Schematic of statistical analysis of the amount of bioelectric potential change monitored by the electrodes.

Figure 4 shows the time courses of bioelectric potentials of potted *Opuntia* hybrid plants obtained by Pt, Ag, and BDD electrodes. Drastic changes of bioelectric potentials were monitored by all electrodes during a finger touch on the *Opuntia* hybrid surface. However, the amount of bioelectric potential change monitored by the BDD electrode is more stable than that of the other electrodes. To investigate the reliability of the monitoring system, statistical analyses were performed as follows: the coefficient of variability (CV) [11] for the signal measured by each electrode was calculated by the mean and the standard deviation (SD) of the amount of bioelectric potential change as shown in Figure 5. The calculated mean and SD of potential change, and CV for the electrodes are summarized in Table 1.

**Table 1:** Mean and SD of potential change evoked by a finger touch on potted *Opuntia* hybrid plants and CV for the potential change measured by each electrode.

Electrodes	Potential Change (mV)		CV (SD/MEAN) (%)
	MEAN	SD	
Pt plate	0.046	0.047	103.1
Ag plate	0.137	0.082	60.1
BDD plate	0.208	0.037	15.3

The CV of BDD plate electrodes were 4–7 times smaller in this detection than Ag or Pt plate electrodes. The difference is due to the electrochemical sensitivity of the electrodes. Recently, BDD electrodes have been shown to have excellent electrochemical sensitivity [6,7,8,9] and have proven suitable for *in vivo* electrochemical detection, such as dopamine generation in the brain [8] or the reduced form of glutathione for the assessment of cancerous tumors [9]. In the present research, the bioelectric potential changes evoked by surface potential of the finger and/or the ion flow through the ion channel of the plant cells [2] were successfully detected by BDD electrodes.

**Figure 6:**
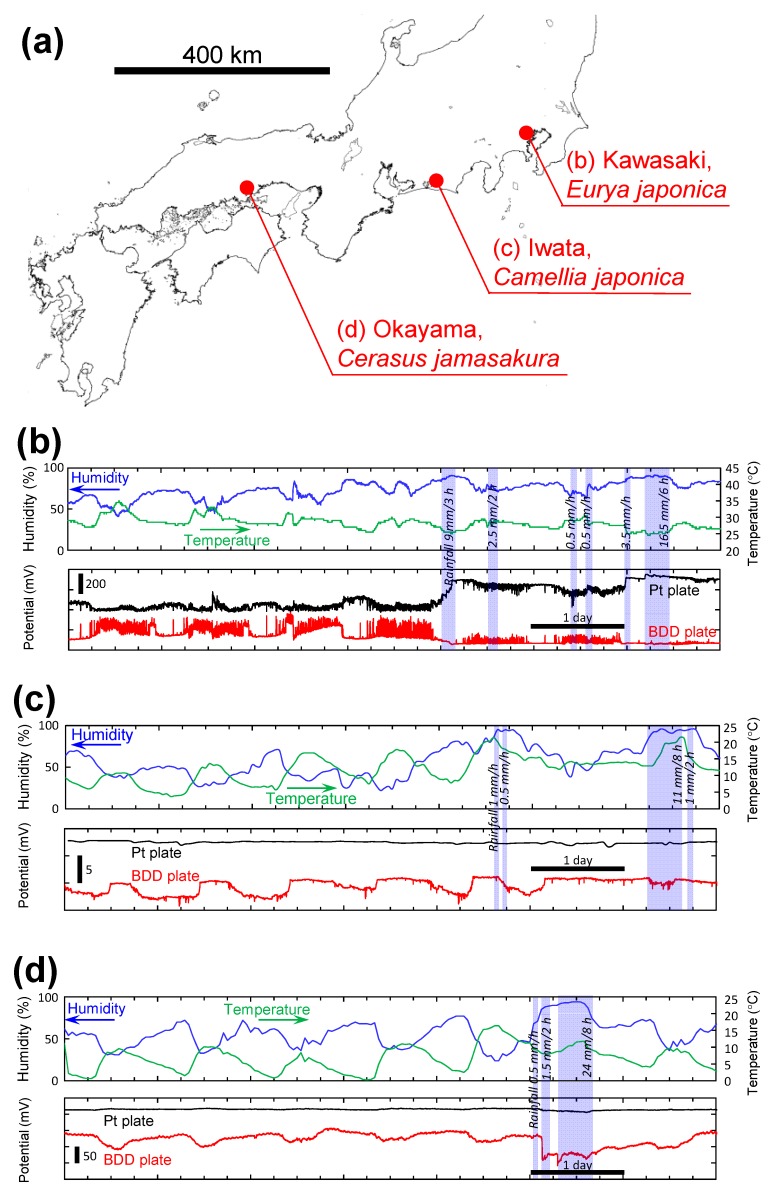
(**a**) Locations and scientific name of the tested ground-planted trees; (**b**–**d**) Schematic of statistical analysis of the amount of bioelectric potential change monitored by Pt and BDD electrodes for the tested ground-planted trees. Temperature, humidity and rainfall data were based on the data published in the website of Japan Meteorological Agency (http://www.data.jma.go.jp/obd/stats/etrn/) at the closest weather monitoring spot to each plant.

A similar tendency was observed in the ground-planted trees. Figure 6a shows the locations and scientific name of the tested trees. We found that both BDD and Pt plate electrodes detected bioelectric potential changes in all the tested trees for months (Figure 6b–d, only a week of the data is shown), BDD plate electrodes were 5–10 times more sensitive in this detection than Pt plate electrodes. For example, for *Eurya japonica* at Kawasaki (Figure 6b), spike-like bioelectric signals can be detected on baseline patterns with high S/B ratio by BDD plate electrodes. These spike-like signals are thought to be the result of transpiration, movement of leaves, and circadian rhythms of plants [5,12]. Interestingly, during rain, although Pt electrodes detected only small baseline shift, BDD electrodes detected a signal of an intensity which is one-half of that obtained while sunny. These results indicated that electrochemical signals in plants changed in response to changing environmental factors such as temperature and humidity as reported in the literature [1,2,3,4,5]. For the other trees, BDD electrodes also detected these signals of plants for a week while Pt electrodes obviously cannot detect them (Figure 6c,d). Thus, BDD electrodes on living plant tissue were able to effectively detect these bioelectrical changes and serve as a monitoring system of the plant glowing and a warning system for impending events such as a storm.

## 4. Conclusions

In this study, a sensitive plant monitoring system based on the detection of bioelectric potentials in plants with BDD electrodes was investigated. The BDD electrodes were 4–7 times more sensitive to bioelectric potential changes in potted *Opuntia* hybrid plants compared to Pt or Ag electrodes. Similarly, for ground-planted trees, BDD electrodes were 5–10 times more sensitive than Pt electrodes in the detection of daily bioelectric potential changes. Given these results, we conclude that BDD electrodes placed on live plant tissue are able to consistently and effectively detect bioelectrical potential changes for a long time and serve as a monitoring system for plant growth and as a warning system for impending environmental events.
